# Ethical failings of CPSO policy and the health care consent act: case review

**DOI:** 10.1186/s12910-019-0357-y

**Published:** 2019-03-19

**Authors:** Joshua T. Landry, Rakesh Patel, David Neilipovitz, Kwadwo Kyeremanteng, Gianni D’Egidio

**Affiliations:** 1 University of Ottawa, Ottawa, ON Canada; 2 Ottawa Hospital Research Institute, Ottawa, ON Canada; 3 Ontario Shores Centre for Mental Health Sciences, Whitby, ON Canada

**Keywords:** End-of-life, Autonomy, Medical consent, Palliative care, Quality of life, Ethical responsibility

## Abstract

End-of-life disputes in Ontario are currently overwhelmingly assessed through the singular lens of patient autonomy. The current dispute resolution mechanism(s) does not adequately consider evidence-based medical guidelines, standards of care, the patient’s best interests, expert opinion, or distributive justice. We discuss two cases adjudicated by the Consent and Capacity board of Ontario that demonstrate the over emphasis on patient autonomy. Current health care policy and the Health Care Consent Act also place emphasis on patient autonomy without considering other ethically defensible factors. We argue that current policy and legislation require amendment, and unless there are measures undertaken to modify them, both the quality of care provided and the long-term capabilities of the health care system to remain publicly-funded, comprehensive and equitable, are at stake.

## Background

The struggle between what *can* versus what *should* be provided to patients, in terms of medical intervention, has led to an increasing number of conflicts between physicians and patients or substitute decision-makers (SDMs).

In the context of the Intensive Care Unit (ICU), a majority of such conflicts involve the withholding or withdrawal of potentially life-prolonging medical treatment, and we argue that the current mechanisms in place for resolving these issues are limited by current policy and law. Specifically, we challenge modifications to the College of Physicians and Surgeons of Ontario (CPSO) Planning for and Providing Quality End-of-Life Care policy [1], in conjunction with a dated Health Care Consent Act (HCCA) [2] that favors patient autonomy over all other elements. We present two cases and arguments recently penned by Bioethicists Julian Savulescu and Peter Singer [3] and apply the three justifications they provide for withholding or withdrawing potentially life-prolonging medical treatments to our current context.

We argue that current policy and legislation require amendment to consider ethically-defensible factors in addition to patient autonomy.

## Main text

### End-of-life decision-making and dispute resolution requires a more clearly defined and ethically-sound foundation

In their recent commentary, Savulescu and Singer [3] argue that,

There are three justifications for withholding or withdrawing potentially life prolonging medical treatment: the patient validly refuses it (autonomy); the expected utility is too low to warrant the public resources necessary to provide it (distributive justice); or it is against the patient’s best interests (interests).

#### On autonomy

Respect for patient autonomy is a central pillar of ethical medical practice. When discussing the rights of autonomous patients, we may acknowledge two different types: positive and negative rights. A positive right would entitle the patient to, or allow them to demand, the provision of a treatment, and a negative right would enable the patient to be free from any unwanted treatment or interference. We take the common view that the negative right of a competent patient ought to be supported, but an absolute positive right to receive whatever treatment the patient demands should not exist. We begin from the starting point that evidence-informed medical opinion is necessary to constrain the options available to patients to ensure they receive ethically-appropriate care that is supported by the best available medical evidence.

#### On distributive justice

When considering which treatments to offer in a publicly-funded health care system, providers must acknowledge, alongside their judgment of a treatment’s effectiveness, benefits, and burdens, that there will always be fewer resources than patients who need them. Because of this, there may be cases in which the expected utility of providing a resource to a particular patient is too low to justify the public resources necessary to provide it. Unfortunately, there is no universally agreed upon method to calculate such utility, despite some methods’ (e.g. a Quality-Adjusted Life-Years approach) recently gaining increased acceptance. These methods are not readily or universally applicable to all patients, at all times, in all contexts.

#### On best interests

Savulescu and Singer [3] note that when a decision is made to withhold or withdraw treatment in the name of a patient’s best interests, this can be justified in three ways:

First, a treatment may be withheld or withdrawn if it is medically futile. The American Thoracic Society (ATS) joint policy statement on *Responding to Requests for Potentially Inappropriate Treatments in Intensive Care Units* endorses a strict definition of futility, where the “intervention simply cannot accomplish the intended physiologic goal.” [4] Despite the appearance of a clear definition, the concept of medical futility has been vigorously contested elsewhere [5–8]. For the purpose of this article, we will assume the first part of a definition as presented by Savulescu and Singer [3], which resembles the aforementioned ATS definition: that a treatment is futile because it has no scientific rationale (e.g. treating a virus with antibiotics). Second, if the expected probability of treatment success is sufficiently low but more than zero, providers must consider other alternatives that may result in a higher probability of success, and what harms or suffering will be differentially incurred. Of course, the limits and variability of a physician’s prognostication must be considered as it relates to this condition, since empirical evidence frequently indicates that physician prognostication is often quite poor. In a study to determine the accuracy of such prognostication, for example, Nicholas Christakis and Elizabeth Lamont found that only 20% of physician estimates of terminally-ill patient survival were accurate within 33% of those patients’ actual survival, when an accurate prediction was defined as between 0.67 and 1.33 times the actual survival. [9] What this would mean, for example, is that if a patient’s actual survival was 30 days after diagnosis, an accurate physician prognosis would be an estimated survival between 20.1 and 39.9 days. This concern requires a more robust review than can be provided in this short article. The third and final way in which best interests may be used to justify withholding or withdrawing treatment is when even successful treatment would leave the patient in a state few would accept. In such cases, treatment need not be offered, and may be withdrawn if currently being provided.

### Current policy and legislation restricts physicians’ ability to provide ethically-defensible medicine because of an unreasonable requirement to uphold respect for patient autonomy

#### College of Physicians and Surgeons of Ontario policy

In 2015, the CPSO revised its “Planning for and Providing Quality End-of-Life Care” policy [1] to require that physicians provide CPR or life-sustaining therapy when requested unless they receive consent to withhold it, even if such provision is not medically indicated. This policy requires that physicians strictly comply with patient demands for life-sustaining therapies. The consequence and reality is that it leaves no room for the other relevant ethical considerations discussed above. In that same policy, the CPSO strongly encourages physicians to seek out a dispute resolution process, which may require an application to the Consent and Capacity Board of Ontario (CCB). The CCB is an independent, quasi-judicial tribunal appointed by the province, with the power to adjudicate end-of-life care disputes between the treating team, patient, and SDMs [10]. The CCB will hear arguments from these parties and base their ruling on the HCCA and other similar legislation.

#### Health care consent act

As it is written, the HCCA requires that SDMs comply with the wishes of the patient on whose behalf they are making a decision, if these wishes are known, applicable to the circumstance, and if it is possible, in the most literal sense, for the wishes to be followed [2]. Below, we will discuss two cases adjudicated by the CCB that demonstrate the shortcomings of the HCCA, with regard to its emphasis on autonomy. In both cases, the treating team was required to offer, or else continue to provide, interventions that were not medically indicated, and that those teams considered harmful.

### What this means in practice: case review

Both DW (Re) 2011 [11] and JEP (Re) 2017 [12] had advanced dementia with multi-organ failure, requiring admission to the intensive care unit during prolonged hospitalizations. Clinical assessments of several intensivists and various specialists suggested that both ongoing and more aggressive interventions were medically futile, had far too low utility, and were not in the patient’s best interests. Moreover, the treating teams, in both cases, argued that ongoing interventions would only result in discomfort and possible harm, and prolong the dying process. In the case of JEP, there also existed questions about whether ongoing interventions would meet an accepted standard of care. The teams sought consent to withhold CPR, not escalate care, and instead pursue a palliative and comfort care approach.

Both JEP (Re) 2017 and DW (Re) 2011 wished to live as long as possible, with no regard for suffering or indignity. In both cases, intractable disagreement caused the health care teams to seek guidance from the CCB. In both cases, the CCB decided that the SDMs were complying with the principles of substitute decision-making, as outlined in the HCCA. For this reason, no permission was granted by the CCB to withdraw current life-prolonging treatments, or to withhold additional potentially life-prolonging measures (e.g. CPR).

JEP had a living will but it did not contain any written advanced directive for medical treatment. JEP had refused to discuss advanced care directives with his family physician. The SDMs for JEP had provided multiple witness statements that JEP in previous actions, behaviours and stories had made it clear he would “want everything done”.

DW had not made a prior capable wish applicable to the proposed treatment plan or about end of life decision-making. The CCB panel reviewed DW’s Power of Attorney for Personal Care and no such prior capable wish was contained in that document. MW (SDM for DW),

Testified that DW had many discussions with her about end of life over the years in many different contexts. She stated that DW did not believe, because of his own interpretation of his religion and because of his general values, in artificially ending a life and that termination of life support or not doing the utmost to remain alive was the equivalent of suicide. She stated that it would be his wish, if he were able to express it, to continue with aggressive treatment [11].

In addition, SW, a daughter of DW claimed that she visited the hospital “almost daily,” and that she was “‘very certain’ that MW’s position about the proposed treatment plan reflected DW’s wishes, values and beliefs” [11]. SW recalled a close friend of the family, “Suzanne”, who suffered from multiple sclerosis, and with whom her father discussed difficult medical choices. It was noted that DW’s advice to Suzanne was always to “‘keep fighting, keep holding on and pray,’ [which] was reflective of DW’s values and beliefs and that he would ‘prefer to suffer’ than to end his life by not pursuing all possible options” [11].

To enforce DW’s beliefs and values, SW added “that she knew that DW was suffering but that despite that, it would be his wish to continue [treatment]. To DW, to induce death in any manner or to forego treatment was ‘mercy killing’” [11]. As well, she emphasized that, irrespective of any pain it may cause, DW would have wanted full resuscitative interventions, since foregoing such measures was, in his view, a sin for which he would go to Hell [11].

To explicate these rulings and understand why they are ethically problematic, we first turn to section 21 of the HCCA.

#### The health care consent act

Section 21 of the HCCA provides an important example of the weight placed on patient autonomy in current provincial legislation. It reads:

- *21. (1) A person who gives or refuses consent to a treatment on an incapable person’s behalf shall do so in accordance with the following principles:*
  1. If the person knows of a wish applicable to the circumstances that the incapable person expressed while capable and after attaining 16 years of age, the person shall give or refuse consent in accordance with the wish.
  2. If the person does not know of a wish applicable to the circumstances that the incapable person expressed while capable and after attaining 16 years of age, or if it is impossible to comply with the wish, the person shall act in the incapable person’s best interests [2].

Here is the greatest limitation with the act; in subsection 1 above, the substitute decision-maker is required to follow the wish of the patient without consideration of other arguably relevant contemporary factors pertinent to the patient’s current medical status. The “wish” need not have been consistently stated by the now incapable patient, nor does it matter that the patient understood, in the sense of informed consent, what they had wished for. Wishes may be expressed in a power of attorney, in a form prescribed by regulations, in any other written form, orally or in any other manner [2]. Moreover, the CCB in its decision-making process are not to consider whether the treatment is medically indicated. There is no regard for evidence-based medicine, including expert or professional opinion and advocacy, and the current best interests of the patient are not considered unless there is no expressed wish or that wish is impossible to comply with. A “wish that is impossible to comply with” refers to whether the physical act of implementing the wish, such as CPR or organ transplant, *can* be done, and not to whether a medical intervention *should* be offered or implemented. In the absence of an explicit wish, the SDM is legally required to consider the incapable person’s best interests; the SDM must contemplate the values and beliefs they know the incapable person held when capable and believe he or she would still act on if capable. Of course, we believe it to be logical to require that SDMs consider the patient’s prior-expressed wishes when making treatment decisions on their behalf. What we contest is that there is not currently an effective mechanism or process to establish limits on what the patient or their SDM could demand, and expect to be provided with in terms of medical intervention.

### Current policy and legislation requires amendment

We propose that it is time for Federal and Provincial governments to address this challenging issue, and we recommend ethically-sound requirements in policy and legislation. Such policy and legislation should be amended to more clearly articulate relevant medical evidence, medical experience and ethical considerations, in addition to the current commitment to respect for patient autonomy, thus ensuring the treatments patients receive are ethically defensible. Although the topic is divisive and uncomfortable, deference to these issues is detrimental to both patients and the publicly-funded health care system. Ideally, leadership and moral suasion from a federal level would be helpful to provide a nudge in this direction.

Our suggestion is to amend section 21 of the HCCA act to include considerations of distributive justice, of patients’ best interests not limited to cases in which patient wishes are not known, and of futility. In addition, we would also advocate that the section contain references to evidence-based medicine and expert opinion; recommending that if the treatment is not indicated, it should not be offered. This point, however, is where CPSO policy and the HCCA offer diverging requirements.

The present CPSO policy compels physicians to offer CPR even when there is no hope for recovery or for ongoing life. Specifically, the policy states that:

A decision regarding a no-CPR order cannot be made unilaterally by the physician […]. If the patient or substitute decision-maker disagrees and insists that CPR be provided, physicians must engage in the conflict resolution process [….]. While the conflict resolution process is underway, […] if an event requiring CPR occurs, physicians must provide CPR unless the patient’s condition will prevent the intended physiologic goals of CPR from being achieved [1].

Consent legislation, on the other hand, does not provide for any treatment to be given by default [13]. In fact, previous rulings have said that providing CPR should be left to the discretion of physicians [14, 15], as informed by the medical evidence.

The current CPSO policy regarding CPR provides patients and their SDM(s) with a positive right: the right to demand and receive an intervention. This is not ethically justifiable and contravenes both the HCCA and the process of informed consent. The former CPSO policy, Decision Making for the End-of-Life, stated that decisions regarding CPR and other potentially life-sustaining treatments should be made according to the likelihood of benefit to the patient and should take into account his or her goals, values, and beliefs [16]. It also noted that Physicians are not obliged to provide treatments that will almost certainly not be of benefit to the patient.

We strongly suggest that the CPSO revise its policy and allow physicians to use their professional judgement, based on the best available medical evidence, on when to offer and provide CPR and other forms of potentially life-sustaining therapy. A treatment should not be provided simply because an individual or their SDM demands it and it can be implemented. Rather, it should only be provided if there is some reasonable probability of benefit*,* if it is desired, and if it can be defended on grounds of distributive justice. We believe that if there is any reasonable doubt about whether a treatment will provide medical benefit to the patient, and our other considerations presented above are met, that it is better to err on providing therapy than not. Although “reasonable doubt” is itself subjective, some clarity and guideline(s) could become a topic of informed debate. In addition, in cases where questions or debate still exist, the CCB would continue to have an important role for adjudication.

The CCB has the responsibility to interpret the HCCA when making a decision regarding consent to treatment and a physician’s proposed plan of care. It is limited in its ability to rule based on current law, and consequently, it cannot adequately address our concerns. Based on the requirements of the HCCA and other legislation, the CCB places excessive weight on the principle of autonomy in its decisions and ignores many of the relevant considerations presented here. As such, any previously-expressed “wish” of an incapable patient, regardless of how uninformed it may be, or whether it is supported by evidence-based medicine, is of paramount and often misplaced importance to current CCB decisions. The amendments to legislation and professional policy recommended above will allow the current CCB model to take into consideration additional relevant factors when making complex decisions, will allow this process to remain responsive and viable, and will support ethical decision-making on behalf of patients who can no longer decide for themselves.

## Conclusions

Based upon our personal experience with CCB hearings in the context of evidence and experience-based critical care medicine, we have highlighted specific flaws in the HCCA, CPSO policy, and CCB process. We believe the principle of autonomy has undue influence on medical decision-making, and that this principle has, in some cases, limited clinician ability to provide ethically-defensible care that is in the patient’s best current interests. Autonomy is a vital component of consent and the derivation and execution of treatment plans; however, it cannot always outweigh or override other germane ethical considerations nor should it provide for a positive right. Unless there are measures undertaken to modify the current CPSO policy or amend the HCCA, the quality of care provided is at stake, as are the long-term capabilities of our health care system to remain publicly-funded, comprehensive and equitable. Revisiting this crucial topic would be of benefit to all of us as patients.

## Abbreviations

ATS: American Thoracic Society
CCB: Consent and Capacity Board
CPR: Cardiopulmonary Resuscitation
CPSO: College of Physicians and Surgeons of Ontario Shores
HCCA: Health Care Consent Act
ICU: Intensive Care Unit
SDM: Substitute Decision-Maker
